# Effectiveness of Acupuncture for Pain Control After Cesarean Delivery: A Randomized Clinical Trial

**DOI:** 10.1001/jamanetworkopen.2022.0517

**Published:** 2022-02-28

**Authors:** Taras I. Usichenko, Berthold Johannes Henkel, Catharina Klausenitz, Thomas Hesse, Guillermo Pierdant, Mike Cummings, Klaus Hahnenkamp

**Affiliations:** 1 Department of Anesthesiology, University Medicine of Greifswald, Greifswald, Germany; 2 Department of Anesthesia, McMaster University, Hamilton, Ontario, Canada; 3 Department of Radiology, University Medicine of Greifswald, Greifswald, Germany; 4 Department of Gynecology and Obstetrics, University Medicine of Greifswald, Greifswald, Germany; 5 British Medical Acupuncture Society, London, United Kingdom

## Abstract

**Importance:**

A pharmacological approach to pain control after cesarean delivery is often insufficient on its own. Acupuncture is a promising method for mitigating postoperative pain and reducing postoperative opioid requirements.

**Objective:**

To evaluate the efficacy and effectiveness of acupuncture as an adjunctive therapy for pain control after cesarean delivery, compared with a placebo intervention and standard care alone.

**Design, Setting, and Participants:**

This single-center, placebo-controlled, patient- and assessor-blinded randomized clinical trial was conducted from January 13, 2015, to June 27, 2018, at a tertiary university hospital in Greifswald, Germany. Participants were women who were scheduled for elective cesarean delivery under spinal anesthesia and were randomized to either the acupuncture group (n = 60) or placebo group (n = 60). Another 60 consecutive patients who met the eligibility criteria and received the standard postoperative analgesia were selected to form a nonrandomized standard care group. The intention-to-treat analysis was performed from August 19, 2019, to September 13, 2019.

**Interventions:**

In addition to standard pain treatment, each patient in the acupuncture group received auricular and body acupuncture with indwelling intradermal needles, whereas patients in the placebo group were treated with nonpenetrating placebo needles.

**Main Outcomes and Measures:**

The primary outcome was pain intensity on movement, which was measured using an 11-item verbal rating scale. Secondary outcomes were analgesia-related adverse effects, analgesics consumption, time to mobilization and Foley catheter removal, quality of patient blinding to randomization, and patient satisfaction with treatment of pain.

**Results:**

A total of 180 female patients (mean [SD] age, 31 [5] years) were included in the intention-to-treat analysis. The mean pain intensity on movement in the acupuncture group on the first postoperative day was lower than in the placebo group (4.7 [1.8] vs 6.0 [2.0] points; Cohen *d*, 0.73; 95% CI, 0.31-1.01; *P* = .001) and the standard care group (6.3 [1.3] points; Cohen *d*, 1.01; 95% CI, 0.63-1.40; *P* < .001). On the first postoperative day, 59 patients (98%) in the acupuncture group were fully mobilized vs 49 patients (83%) in the placebo group (relative risk [RR], 1.18; 95% CI, 1.06-1.33; *P* = .01) and 35 patients (58%) in the standard care group (RR, 1.69; 95% CI, 1.36-2.09; *P* < .001). The Foley catheter was removed in a total of 57 patients (93%) from the acupuncture group vs 43 patients (72%) from the placebo group (RR, 1.33; 95% CI, 1.12-1.57; *P* = .003) and 42 patients (70%) from the standard care group (RR, 1.37; 95% CI, 1.14-1.62; *P* = .002). Other parameters were comparable across the 3 study groups.

**Conclusions and Relevance:**

Results of this trial showed that acupuncture was safe and effective in reducing pain and accelerating mobilization of patients after cesarean delivery. With consideration for personnel and time expenditures, acupuncture can be recommended as routine, supplemental therapy for pain control in patients after elective cesarean delivery.

**Trial Registration:**

ClinicalTrials.gov Identifier: NCT02364167

## Key Points

**Question:**

Is adding preoperative acupuncture to standard pharmacological therapy effective for pain control in patients after cesarean delivery?

**Findings:**

In this randomized clinical trial with 180 female patients, acupuncture reduced pain on movement and accelerated patient mobilization after cesarean delivery compared with placebo acupuncture and standard pharmacological therapy alone.

**Meaning:**

Findings from this trial suggest that use of preoperative acupuncture as an additional pain therapy is safe and effective in patients after elective cesarean birth.

## Teaser

This randomized clinical trial examines the safety and efficacy of acupuncture as a supplemental preoperative treatment for pain in women who will undergo cesarean birth.

## Introduction

Better understanding of the pathophysiological and pharmacological components of nociception and the implementation of procedure-specific, multimodal analgesic pathways have led to improved quality of treatment for acute postoperative pain after various surgical procedures.^1,2,3^ However, patients who have undergone cesarean delivery still experience high levels of postoperative pain, which may be attributed to insufficient use of opioid analgesics.^4,5^ The pharmacological treatment of postoperative pain after cesarean delivery is often restricted because of the priorities of childcare and breastfeeding,^6,7^ and only a few analgesic drugs are therefore recommended for such postoperative pain.^6,7,8^ A systematic review of randomized clinical trials (RCTs) on oral analgesia for post–cesarean birth pain found that none of the 13 included studies reported adequate pain relief.^9^ Given these previous findings and attempts to decrease the high prescription rate of postoperative opioid analgesics,^10,11^ it is reasonable to consider the use of nonpharmacological methods in adjunctive treatment of postoperative pain. Acupuncture is one such method.

Acupuncture allows reduced use of postoperative opioid analgesics, intensity of postoperative pain, and incidence of opioid-related adverse effects.^12,13,14^ For the treatment of post–cesarean delivery pain, acupuncture has been associated with decreased postoperative pain intensity and analgesics dose as well as increased patient satisfaction in several preliminary reports.^15,16,17^ Informed by the needling methods summarized by Sun et al,^12^ we developed and tested a combined body and ear acupuncture method for the treatment of postoperative pain in a series of patients who were scheduled for elective cesarean delivery.^17^ This method was well accepted, and the outcomes allowed the estimation of sample size for a subsequent RCT.^17^ The aim of the present investigation was to evaluate the efficacy and effectiveness of acupuncture as an adjunctive therapy for pain control after cesarean delivery compared with a placebo intervention and standard care alone.

## Methods

This single-center, placebo-controlled, patient- and assessor-blinded RCT with an additional nonrandomized control (standard care) group was performed at the Department of Gynecology and Obstetrics at the University Medicine of Greifswald in Greifswald, Germany, between January 13, 2015, and June 27, 2018. All participants provided written informed consent. The study was carried out in accordance with the principles of the Declaration of Helsinki^18^ and was prospectively approved by the institutional ethics committee of the University Medicine of Greifswald. We followed the Consolidated Standards of Reporting Trials (CONSORT) reporting guideline. The trial protocol is available in Supplement 1.

We included consecutive adult female patients with an American Society of Anesthesiologists physical status of II to III who were scheduled to undergo an elective Misgav Ladach method for cesarean delivery under spinal anesthesia. Patients who had a history of alcohol abuse, were using opioids or psychotropic medication, or were unable to understand the informed consent form and/or fill out the study questionnaire were excluded. Additional exclusion criteria after enrollment included failure of spinal anesthesia, cesarean delivery duration longer than 60 minutes, intraoperative complications, and neonatal complications.

### Randomization and Group Allocation

**Figure 1.**
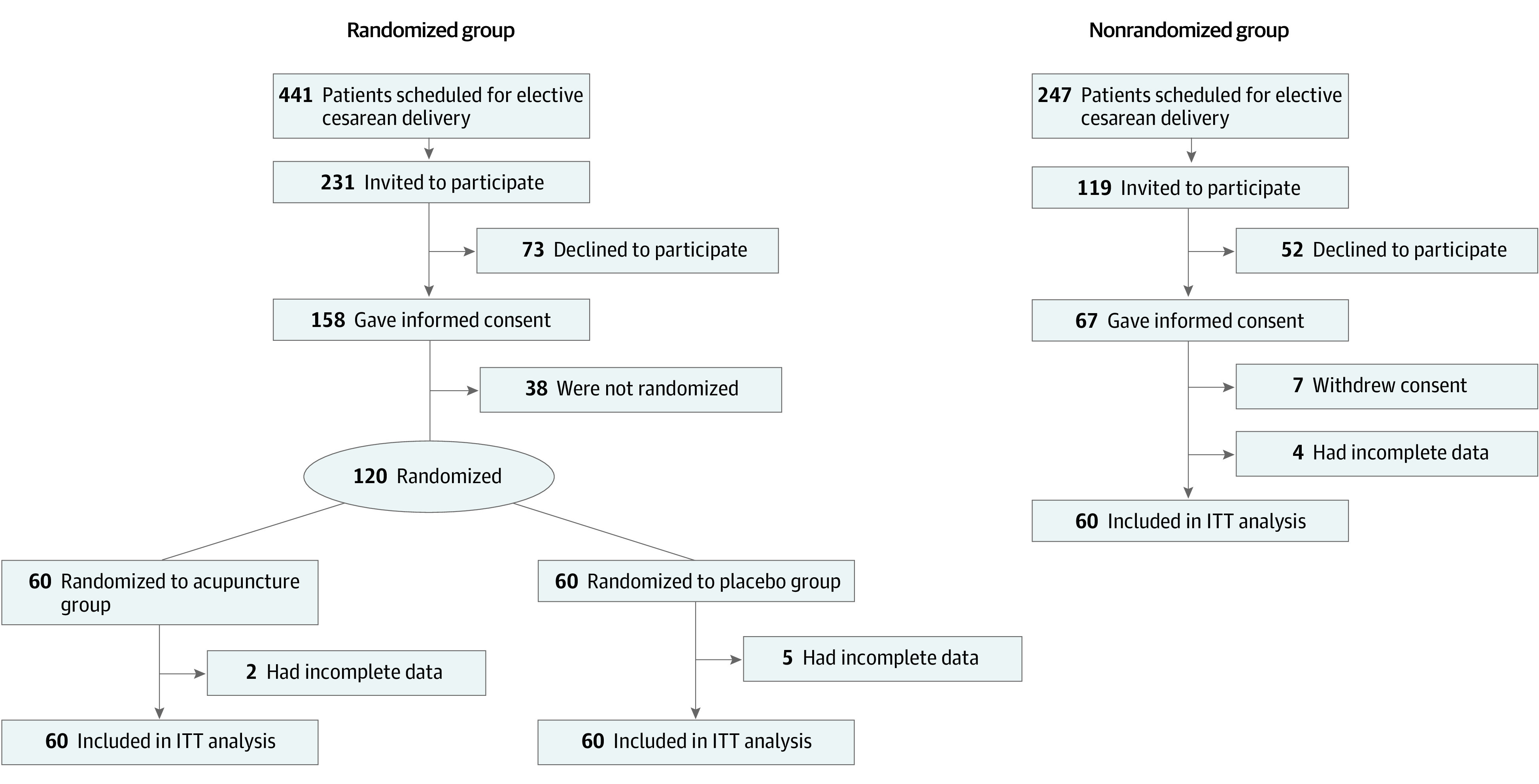
CONSORT Diagram ITT indicates intention-to-treat.

During the standard preoperative examination, eligible patients were invited to participate in the RCT wherein they would receive either acupuncture or placebo acupuncture in addition to standard postoperative pain treatment after cesarean delivery. Patients who agreed to participate and signed an informed consent form were randomized to either the acupuncture or placebo treatment (Figure 1) on the day of cesarean delivery before spinal anesthesia was administered.

One of 3 physicians with experience in acupuncture (T.I.U., C.K., or T.H.) provided the acupuncture or the placebo intervention and performed the randomization using the sealed envelope method. The envelopes, each of which contained a piece of paper on which either “AKUPUNKTUR” or “PLACEBO” was typewritten, were opened by the physician to find out which procedure to perform. The physician attached a sticker with the patient’s name and date of birth to this piece of paper and then sealed the envelope again. The envelopes were reopened, and group allocation data were extracted and added to the main data table after the statistical analysis was finished. With this process, randomization was concealed from patients, assessors of the outcome, and practitioners who provided care during and after the cesarean delivery. Only the physicians who performed the study interventions were aware of the group allocation, although they had contact with the participants after the intervention was completed. The first patient who was randomized was included on January 13, 2015, and the last patient was included on June 7, 2017.

Patients who met the eligibility criteria but were not included in the randomized investigation were invited to participate in a routine audit of quality management system in the treatment of acute postoperative pain. These patients constituted the third nonrandomized group, which received standard care without additional intervention (standard care group). The last patient for the standard care group was recruited on June 27, 2018.

### Interventions

According to the trial protocol, all of the interventions were performed by at least 2 of 3 physicians (T.I.U., C.K., T.H.) immediately after randomization in the patients’ ward and were completed within 20 minutes before the cesarean delivery. During the intervention, 1 of the physicians provided the patient with structured information on the (1) potential benefits of acupuncture, (2) potential mechanisms of acupuncture, (3) potential adverse effects, and (4) postoperative handling of semipermanent indwelling needles.

#### Acupuncture

While a physician was providing an overview of the intervention to the patient, the second physician was performing the bilateral needling of 4 auricular acupuncture points and 6 body acupuncture points, according to the trial protocol. For auricular acupuncture, indwelling fixed needles (New Pyonex; Seirin Corp) were used, which were 1.5 mm in length and 0.2 mm in diameter. The indwelling fixed needles are tiny needles that are embedded in a small plastic hemispherical knob within a disc of flesh-colored self-adhesive tape. For body acupuncture, we used intradermal needles (Spinex; Seirin Corp), which were 6 mm in length and 0.14 mm in diameter, that were covered and secured using placebo New Pyonex needles. Thus, these needles resembled auricular acupuncture needles but consisted of only the plastic knob and self-adhesive tape without needles, appearing identical to patients in the placebo group.^17^ Details and pictures of the equipment used for the interventions, the list of specific acupuncture points, and the rationale for their choice are published elsewhere.^17^

The physicians instructed patients to stimulate the auricular needles and LI4 pressure points by massage for 3 to 5 minutes before asking for additional analgesic medication if they experienced pain. This pain had to have an intensity higher than 4 points on an 11-item verbal rating scale (VRS-11), where 0 points indicated no pain and 10 points indicated maximal pain.

#### Placebo Acupuncture

For patients in the placebo group, the second physician attached the placebo needles near the specific acupuncture points. To imitate the pricking sensation of real needles during the application of placebo needles, the physician examined the skin areas around acupuncture points with a SVESA neural pen (Neuralstift SVESA 1070; SVESA), which is commonly used in acupuncture practice to identify skin areas with lower skin resistance.^19^ The SVESA neural pen has a thin tip that produces the feeling of needle insertion if certain pressure is applied to the skin. The physician informed the patient that the neural pen was being used to find the acupuncture point, but the pen was actually being used to produce a pressure with the tip.

Both acupuncture needles and placebo needles were placed before spinal anesthesia was administered. The needles remained in situ for 3 days after cesarean delivery.

### Standard Care

Immediately following the application of acupuncture or placebo needles, patients were transferred to the operating room where they received standardized spinal anesthesia consisting of 7.5 mg hyperbaric bupivacaine hydrochloride and 5 μg sufentanil citrate before cesarean delivery. The combination of bupivacaine and sufentanil provides sufficient analgesia for a maximum of 4 hours after the surgery. Standard postoperative pain treatment was provided according to local clinical guidelines.^5^ Postoperative analgesia consisted of 1 g oral paracetamol 4 times a day and supplemented by 50 mg diclofenac potassium 3 times a day, if necessary. In case of insufficient analgesia, subcutaneous injections of 7.5 mg piritramide (an opioid analgesic with 70% of the potency of morphine) were allowed up to 6 times a day.

### Study End Points and Sample Size Calculation

Study end points were evaluated with a previously validated questionnaire that was based on the Brief Pain Inventory (Supplement 1).^20^ The primary end point was pain intensity on movement, which was measured on the first postoperative day using the VRS-11. Secondary end points were the intensity of maximal and minimal pain on the first postoperative day and the intensity of pain on movement on the day of discharge; incidence of analgesic adverse effects (including nausea, vomiting, and/or tiredness); disturbance of movement, mood, sleep, and/or enjoyment of life by pain; total consumption of paracetamol, diclofenac, and piritramide during the postoperative period; and time to mobilization (sitting, standing up at the bed, and visiting the lavatory or ambulation) and removal of the Foley catheter after cesarean delivery (eTable 2 in Supplement 2).

The decision to start or continue mobilization was made after a clinical assessment by a nurse. The decision to remove the Foley catheter was also made by a nurse on the basis of whether the patient could visit the lavatory (or ambulate). On the day of discharge, patient satisfaction with treatment of pain after cesarean delivery was assessed using a 5-item VRS, where 1 point indicated excellent and 5 points indicated very bad. The quality of patient blinding (to randomization to either acupuncture or placebo group) was also tested. Moreover, the patients were asked whether, in the future, they would like to receive acupuncture for pain control after cesarean delivery. To assess the generalizability of the results, one of us (B.J.H., who was responsible for collection of other study end points) recorded self-reported race and ethnicity. All participants identified as being of non-Hispanic White race and ethnicity.

Data on pain intensity on movement on the first postoperative day from the clinical audit^5^ (mean of 5.5 points on the VRS-11) and from the pilot investigation^17^ (mean of 4.1 points) were used for a sample size calculation in this RCT. Assuming the variability in pain intensity on movement on the first postoperative day of 2.0 SD, 55 patients per group were needed to demonstrate a difference between groups of 1.4 at a significance level (type I error rate) of .05 and power of 90%, taking into account a Bonferroni adjustment for multiple comparisons between the acupuncture, placebo, and standard care groups. To account for an expected dropout rate of 10%, the size of these groups was inflated to 60 patients each.

### Statistical Analysis

The intention-to-treat analysis was performed from August 19, 2019, to September 13, 2019. Normally distributed continuous data were compared using an unpaired, 2-tailed *t* test. Skewed data were compared using the Mann-Whitney test. The Fischer exact test was used to analyze the dichotomous data. A 2-sided *P* < .05, which was Bonferroni-adjusted for multiple comparisons as appropriate, was considered to be statistically significant. The effect size for continuous variables was calculated using the standardized mean difference (Cohen *d*), and the effect size for dichotomous variables was calculated as relative risk (ie, ratio of patients in the treatment group who improved divided by the percentage of patients in the control [standard care] group who improved).

Statistical analysis was performed with the SPSS for Mac, version 22.0 (SPSS Inc) and QuickCalcs (GraphPad). Data are presented as mean (SD) and number (percentage) of patients, unless otherwise stated.

## Results

Of 441 patients who were scheduled for elective cesarean delivery during the study period, 231 were invited to participate in the RCT, 73 declined to participate (Figure 1), and 8 were not included because their cesarean delivery was rescheduled and we were not informed. A total of 120 patients were randomized to either the acupuncture group (n = 60) or placebo group (n = 60) (Figure 1). Sixty patients were selected for the nonrandomized standard care group from 247 patients who met the inclusion criteria but were not included in the randomized investigation. All 180 participants were women, had a mean (SD) age of 31 (5) years, and identified as White individuals. Demographic characteristics and relevant intraoperative parameters were comparable across all 3 study groups (Table 1).

**Table 1.** Baseline Characteristics of Patients, by Group Allocation

Characteristic	No. (%)		
	Acupuncture group	Placebo group	Standard care group
Patients, No.	60	60	60
Age, mean (SD), y	31 (5)	31 (5)	32 (5)
Weight, mean (SD), kg	85 (16)	87 (20)	87 (20)
BMI, mean (SD)	30.4 (5.1)	29.9 (6.2)	30.8 (6.8)
ASA physical status			
II	55 (92)	55 (92)	52 (87)
III	5 (8)	5 (8)	8 (13)
Multiple pregnancies	4 (7)	2 (3)	3 (5)
No. of previous deliveries			
0	21 (35)	27 (45)	16 (27)
1	25 (43)	22 (37)	27 (45)
2	9 (15)	8 (13)	9 (15)
3	4 (7)	3 (5)	6 (10)
4	1 (2)	0	2 (3)
No. of previous cesarean deliveries			
0	32 (53)	37 (62)	30 (50)
1	20 (33)	22 (37)	22 (37)
2	7 (12)	1 (2)	6 (10)
3	1 (2)	0	2 (3)
Weight of newborn, mean (SD), g	3562 (500)	3439 (614)	3489 (638)
Type of anesthesia			
Spinal	60 (100)	59 (98)	60 (100)
General	0	1 (2)	0
Duration of cesarean delivery, mean (SD), min	36 (10)	36 (18)	39 (14)
Decrease of blood pressure^a^	47 (78)	41 (68)	41 (68)
Administration			
Vasopressor	45 (75)	41 (68)	37 (62)
Atropine sulfate	21 (35)	13 (22)	12 (20)

Abbreviations: ASA, American Society of Anesthesiologists; BMI, body mass index (calculated as weight in kilograms divided by height in meters squared).

^a^ Decrease of blood pressure was defined as blood pressure reduction of at least 20% from baseline.

Despite several cases with incomplete data, the records of 180 patients were included into the intention-to-treat analysis. The rate of missing data for the end points of this RCT did not exceed 8% (eTable 1 in Supplement 2). Cesarean delivery initially started at 8 am, but this starting time was later (on January 5, 2016) moved to 2 pm for all patients.

### Postoperative Pain

On the first postoperative day, patients in the acupuncture group reported lower mean (SD) pain intensity on movement as measured with the VRS-11 compared with patients in the placebo group (4.7 [1.8] vs 6.0 [2.0] points; Cohen *d*, 0.73; 95% CI, 0.31-1.01; *P* = .001) and the standard care group (6.3 [1.3] points; Cohen *d*, 1.01; 95% CI, 0.63-1.40; *P* < .001) (Table 2). Mean (SD) pain intensity on movement was comparable across the 3 groups on both postoperative days (Table 2). Mean (SD) maximal and minimal pain intensity was lower in the acupuncture group than in the placebo group and standard care group, but none of these in-between group comparisons reached statistical significance (Table 2).

**Table 2.** Comparison of Results Among the Acupuncture, Placebo, and Standard Care Groups^a^

End point	Mean (SD)			Acupuncture group vs placebo group		Acupuncture group vs standard care group		Placebo group vs standard care group	
	Acupuncture group	Placebo group	Standard care group	Effect size (95% CI)	*P* value	Effect size (95% CI)	*P* value	Effect size (95% CI)	*P* value
Maximal pain intensity, points^b^^,^^c^	7.1 (1.8)	7.4 (1.6)	7.7 (1.3)	−0.16 (−0.52 to 0.21)	>.99	−0.33 (−0.70 to 0.02)	.12	0.19 (−0.17 to 0.56)	.80
Minimal pain intensity, points^b^^,^^c^	2.2 (1.5)	2.4 (1.9)	2.7 (1.8)	0.11 (−0.48 to 0.26)	>.99	−0.33 (−0.69 to 0.03)	.30	−0.19 (−0.56 to 0.17)	>.99
Pain intensity on movement, points^b^^,^^c^									
First postoperative day	4.7 (1.8)	6.0 (2.0)	6.3 (1.3)	0.73 (0.31 to 1.01)	.001	1.01 (0.64 to 1.40)	<.001	−0.15 (−0.51 to 0.22)	>.99
Day of discharge	2.9 (1.6)	3.2 (1.5)	3.5 (1.4)	0.23 (−0.59 to 0.15)	.90	−0.44 (−0.82 to −0.08)	.09	−0.24 (−0.60 to 0.13)	.80
Pain disturbance^d^									
Movement	51 (87)	52 (88)	53 (90)	0.93 (0.83 to 1.04)	>.99	0.93 (0.82 to 1.04)	>.99	0.99 (0.91 to 1.10	>.99
Mood	25 (42)	26 (44)	29 (48)	0.91 (0.61 to 1.37)	>.99	0.83 (0.56 to 1.23)	>.99	0.91 (0.63 to 1.33)	>.99
Sleep	43 (73)	43 (73)	37 (62)	0.95 (0.77 to 1.17)	>.99	1.12 (0.88 to 1.43)	>.99	1.18 (0.94 to 1.49)	>.99
Enjoyment of life	43 (73)	43 (73)	41 (68)	0.95 (0.77 to 1.17)	>.99	1.01 (0.81 to 1.26)	>.99	1.06 (0.87 to 1.32)	>.99
Total acetaminophen dose, g^c^	8.5 (2.4)	8.7 (1.5)	8.3 (1.7)	−0.11 (−048 to 0.25)	>.99	0.08 (−0.27 to 0.44)	>.99	0.25 (−0.12 to 0.61)	>.99
Total diclofenac potassium dose, median (IQR), mg^c^	50 (50-100)	50 (50-100)	100 (50-150)	0.30 (0.04 to 0.77)	.33	0.01 (−0.34 to 0.36)	>.99	−0.39 (−0.7 to −0.02)	.18
Required piritramide, No. (%)^d^	7 (12)	6 (10)	12 (20)	1.21 (0.43 to 3.37)	>.99	0.60 (0.26 to 1.42)	.88	0.50 (0.20 to 1.25)	.63
Analgesia-related adverse effects, No. (%)^d^									
Nausea	10 (17)	5 (9)	16 (27)	1.85 (0.67 to 5.12)	.29	0.63 (0.27 to 1.21)	>.99	0.33 (0.13 to 0.79)	.18
Vomiting	2 (3)	2 (3)	4 (7)	0.98 (0.14 to 6.63)	>.99	0.50 (0.09 to 2.63)	>.99	0.52 (0.09 to 2.71)	>.99
Tiredness	40 (67)	38 (65)	40 (67)	1.02 (0.79 to 1.30)	>.99	1.03 (0.81 to 1.33)	>.99	1.02 (0.79 to 1.31)	>.99
Patient satisfaction with pain treatment, points^c^^,^^e^	2.0 (0.7)	2.1 (0.7)	2.0 (0.7)	−0.08 (−0.47 to 0.27)	>.99	0.10 (−0.29 to 0.44)	>.99	0.18 (−0.19 to 0.54)	>.99
Duration of hospital stay, d^c^	3.8 (0.8)	3.8 (0.9)	3.6 (0.9)	−0.03 (−0.39 to 0.32)	>.99	0.07 11 (−0.25 to 0.47)	>.99	0.14 (−0.22 to 0.50)	>.99

^a^ Statistical significance was calculated with *t* test, Mann-Whitney test, or Fisher exact test, as appropriate, after Bonferroni adjustment.

^b^ Assessed with an 11-item verbal rating scale (VRS-11), where 0 points indicated no pain and 10 points indicated maximal pain.

^c^ Effect size was calculated with Cohen *d* for continuous variables.

^d^ Effect size was calculated as relative risk for dichotomous data.

^e^ Assessed with a 5-item VRS, where 1 point indicated excellent and 5 points indicated very bad.

The quantity of paracetamol as the standard pain killer and diclofenac as a supplement was comparable among the patients in all 3 groups. Twelve patients (20%) from the standard care group required rescue opioid analgesic piritramide vs 7 patients (12%) from the acupuncture group and 6 patients (10%) from the placebo group, but these differences were not statistically significant (Table 2). Patients in all 3 groups were equally satisfied with the postoperative pain treatment (mean [SD] ranging from 2.0 [0.7] to 2.1 [0.7] points). The mean duration of hospital stay did not differ across the 3 groups (<4 days) (Table 2).

### Postoperative Mobilization

**Figure 2.**
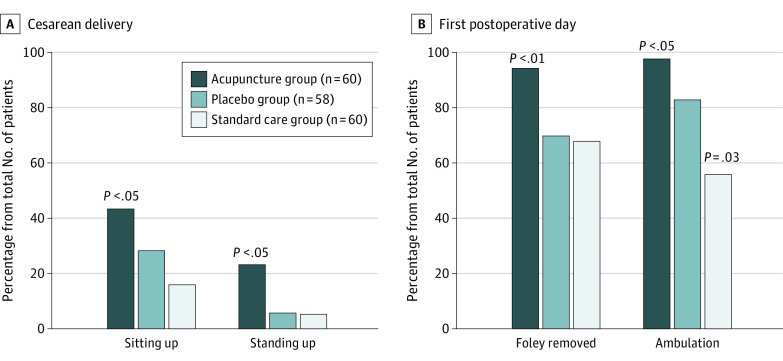
Results of Patient Mobilization on the Day of Cesarean Delivery and on the First Postoperative Day

Forty-one patients (68%) from the acupuncture group vs 19 patients (32%) from the placebo group (RR, 2.13; 95% CI, 1.43-3.25; *P* = .003) and 12 patients (20%) from the standard care group (RR, 3.58; 95% CI, 2.01-5.83; *P* < .001) started their mobilization on the day of cesarean delivery (Figure 2; eTable 2 in Supplement 2). Fifty-nine patients (98%) from the acupuncture group were fully mobilized in comparison with 49 patients (83%) from the placebo group (RR, 1.18; 95% CI, 1.06-1.33; *P* = .01) and 35 patients (58%) from the standard care group (RR, 1.69; 95% CI, 1.36-2.09; *P* < .001) on the first postoperative day; there was a difference between the placebo group and the standard care group (RR, 1.43; 95% CI, 1.09-1.79; *P* = .03). By the first postoperative day, the Foley catheter was removed in 57 patients (93%) from the acupuncture group vs 43 patients (72%) from the placebo group (RR, 1.33; 95% CI, 1.12-1.57; *P* = .003) and 42 patients (70%) from the standard care group (RR, 1.37; 95% CI, 1.14-1.62; *P* = .002) (Figure 2; eTable 2 in Supplement 2).

### Adverse Events and Quality of Life

Forty patients (67%) in either the acupuncture group or the standard care group and 38 patients (65%) in the placebo group complained of tiredness in the postoperative period. In the acupuncture group, 10 patients (17%) had nausea and 2 patients (3%) had vomiting; in the placebo group, 5 patients (9%) had nausea and 2 patients (3%) had vomiting; and in the standard care group, 16 patients (27%) had nausea and 4 patients (7%) had vomiting (Table 2). The incidence of these analgesia-related adverse effects was comparable among patients from all 3 study groups. Postoperative pain interfered with the quality of life in 42% to 90% of patients (Table 2); the incidence of disturbance in aspects of quality of life (movement, mood, sleep, and enjoyment of life) was comparable across the study groups.

Two patients from the acupuncture group complained about unpleasant sensations at the acupuncture sites: 1 patient removed the needles from LI4 points but wished to receive acupuncture for postoperative pain control in the future, whereas the other patient tolerated the needles but did not wish to receive acupuncture in the future. One patient developed bradycardia and hypotension during the placebo intervention after the parturient woman was informed about such adverse effects of acupuncture. The incident was immediately treated without consequences for mother and child,^21^ and the data for this patient were included in the intention-to-treat analysis. All other patients tolerated the acupuncture and placebo intervention well. The occurrence of bradycardia during cesarean delivery was comparable across the 3 groups: 21 patients from the acupuncture group, 13 patients from the placebo group, and 12 patients from the standard care group.

### Quality of Blinding

Twenty-five patients (43%) in the acupuncture group vs 11 patients (20%) in the placebo group believed that they received verum acupuncture, whereas 26 patients (45%) in the acupuncture group and 32 patients (58%) in the placebo group could not identify their group assignment (Table 3). These differences were not statistically significant. Forty-five patients (76%) from the acupuncture group and 48 patients (87%) from the placebo group stated that they would readily receive acupuncture again for additional postoperative analgesia in the future.

**Table 3.** Patients’ Opinion on Acupuncture

	No. (%)		*P* value^a^
	Acupuncture group (n = 58)	Placebo group (n = 55)	
Perception of group allocation			
Real acupuncture	25 (43)	11 (20)	.08
Placebo	7 (12)	12 (22)	.32
Do not know	26 (45)	32 (58)	.52
Do you want acupuncture again?			
Yes	45 (76)	48 (87)	.68
No	14 (24)	7 (13)	.24

^a^ Statistical significance was calculated with Fisher exact test.

## Discussion

In this single-center RCT with randomized acupuncture and placebo groups and nonrandomized standard care group without intervention, we found that acupuncture with indwelling fixed needles for additional analgesia in patients after cesarean delivery was safe and effective in treating postoperative pain and accelerating mobilization compared with placebo and standard therapy alone. Although the clinical effect of acupuncture on postoperative pain intensity on movement was moderate in comparison with placebo (Cohen *d*, 0.73) and was large in comparison with standard care alone (Cohen *d*, 1.01), the mean pain intensity on movement in patients from the acupuncture group was 4.7 points, thus exceeding the desired value of less than 4 points, which is considered on the VRS-11 as an adequate level for postoperative analgesia and patient satisfaction with postoperative pain treatment.^4,22,23^ Acupuncture may be recommended for routine use as supplemental pain therapy for patients after elective cesarean delivery, with consideration for required personnel and time expenditures.

This unexpectedly high level (4.7 points) of pain intensity on movement in the acupuncture group can be explained by (1) the weak-to-moderate analgesic effect of acupuncture in case of acute pain^12,13^ and (2) the accelerated mobilization of patients who received acupuncture; a total of 70% of patients from the acupuncture group started their mobilization on the cesarean delivery day vs 32% of patients from the placebo group and a total of 19% of patients from the standard care group. Meanwhile, it is well known that the recovery priorities of new mothers after cesarean delivery are centered not on pain relief alone but also on mobilization and fast return to everyday activities, including caregiving and breastfeeding.^24,25^

We speculated that the patients in this investigation used the analgesic effect of acupuncture stimulation to regain mobilization for the everyday activity of taking care of their children, while simultaneously taking into account higher (but individually still acceptable) levels of pain. This finding may explain the reports from previous studies of high levels of pain in women after elective cesarean delivery, whereby the intensity of postoperative pain on movement was approximately 6 points (VRS-11) despite the use of various strategies, such as multimodal postoperative pain treatment,^5,7^ on-label and off-label nonsteroidal anti-inflammatory drugs,^8,9^ and opioid analgesics.^26,27^ Such pain reduced quality of life and decreased patient satisfaction with postoperative pain treatment.^4,22,23^

We tested acupuncture in conjunction with short-acting opioid sufentanil, which is commonly used in Germany for intrathecal application for postoperative analgesia in patients.^28^ Recently, intrathecal morphine was recommended as the criterion standard that provides a minimum of 12 hours of sufficient analgesia in patients after cesarean delivery.^29^ Thus, we cannot be sure of the value of acupuncture when long-acting intrathecal opioids are used. Nevertheless, high levels of pain (>6 of 10 points on the VRS-11) have persisted longer than 24 hours after cesarean delivery,^4,5,17^ suggesting the necessity of further investigation of the combined acupuncture and intrathecal morphine for spinal anesthesia. Moreover, regarding the justified concerns of opioid overuse in the perioperative setting,^30^ adding acupuncture to nonopioid medications might be an alternative to using systemic opioid medication in patients who are scheduled for elective cesarean delivery.^15^

We believe the findings of this RCT support the results of previous prospective-controlled investigations on the treatment of acute pain in patients after cesarean delivery.^5,16,31^ The mean pain intensity on movement of 4.7 points in patients who received acupuncture was comparable to that in patients from the pilot study^5^ and the RCT of Wu et al^16^; however, both of these previous studies did not record the mobilization parameters of patients. One RCT that involved 56 patients who were scheduled for cesarean delivery under spinal anesthesia and were randomized either to a single session of acupuncture or a sham procedure did not report any benefits of acupuncture over the sham intervention.^32^ However, a single session of 20-minute needling of 2 acupoints without stimulation suggests that the acupuncture dose was likely insufficient. The application of indwelling intradermal needles in this RCT may have provided an adequate ongoing acupuncture dose through the continuous stimulation of Aβ, Aδ, and/or C afferent fibers from skin and transmission via the spinal ventrolateral funiculus to the brain nuclei, which is considered a leading analgesic mechanism of acupuncture.^33^

We believe that we could enhance the effect of peripheral stimulation of sensory afferents by the stimulation of cranial nerves, especially the auricular branch of the vagus nerve, which is currently considered the mechanism behind the analgesic effect of auricular acupuncture.^34^ This theory is in agreement with the experimental data of Komisaruk and Sansone,^35^ who demonstrated that the upper part of the vagina and the lower part of the uterus receive afferent vagal innervation and suggested the neurophysiological basis for a novel pain-blocking mechanism.^36^ This idea is supported to some degree by the findings of the present trial, in which hemodynamically relevant bradycardia occurred in patients from the acupuncture, placebo, and standard care groups during cesarean delivery. This adverse event required treatment using an antagonist of muscarinic acetylcholine receptors (atropine) to counteract the hypothetical stimulation of parasympathetic nerves from needling in the territory of the auricular branch of the vagus nerve. Beyond this clinically relevant finding, the use of indwelling acupuncture needles, which remained in situ for 3 days after cesarean delivery, was not associated with any adverse effects in this RCT. The safety of using indwelling intradermal needles has been thoroughly discussed in previous studies.^17^

### Limitations

This study has several limitations. First, because the waiting list option for the control group with standard therapy alone, which is commonly used in clinical acupuncture research,^37,38^ was not possible for randomization in a perioperative setting and because all of the patients who were randomized but did not receive the intervention tended to refuse further participation and thus prolonged the RCT, we recruited patients for the nonrandomized standard care group. The use of a nonrandomized group may have introduced some bias and limited the validity of the data.^39^

Second, because of the single-center design of the trial, the recruitment was limited to patients with White race and ethnicity. White individuals are known to demonstrate the highest tolerance for experimental pain and lowest postoperative pain intensity.^40,41^ This aspect might limit the generalizability of the findings; however, theoretically higher baseline levels of pain intensity (taken as the primary outcome measure) may have a greater chance of being influenced by analgesic interventions and may produce substantial change. Third, despite the successful blinding of patients and study personnel who were involved in the treatment of patients and the outcome evaluation, performance bias could have been introduced because of the inability to blind the practitioners (acupuncturists).

## Conclusions

This RCT found that preoperative acupuncture for additional postoperative analgesia was safe, had a clinically relevant effect on pain, and accelerated the mobilization of patients after cesarean delivery without adverse effects within a particular perioperative setting. With additional consideration of personnel and time expenditure, acupuncture can be recommended for routine use as supplemental therapy for pain control in patients after elective cesarean delivery.

## Notes

Supplement 1.
Trial Protocol

Supplement 2. **eTable 1.** Missing Data Across the Study Groups and End Points **eTable 2.** Mobilization and Foley Catheter Removal

Supplement 3.
Data Sharing Statement
