# Errors in Results and Table 3

**DOI:** 10.1001/jamanetworkopen.2022.9622

**Published:** 2022-04-13

In the Original Investigation titled “Effectiveness of Acupuncture for Pain Control After Cesarean Delivery: A Randomized Clinical Trial,”^1^ published February 28, 2022, there were errors in the Quality of Blinding subsection and the *P* values reported in Table 3. In terms of identifying their group assignment, the difference between the acupuncture and placebo groups was statistically significant (*P* = .02). In Table 3 for the perception of group allocation, there were 58 participants in the acupuncture group and 55 in the placebo group. The correct *P* value for the responses is *P* = .02. A total of 59 participants in the acupuncture group and 55 in the placebo group responded to the question, “Do you want acupuncture again?” The correct *P* value for the responses is *P* = .15. This article has been corrected.^1^
