# Supplementary material for: Effectiveness of Acupuncture for Pain Control After Cesarean Delivery: A Randomized Clinical Trial
Source: JAMA Netw Open. 2022 Feb 28;5(2):e220517. doi: 10.1001/jamanetworkopen.2022.0517 (PMC8886541; doi:10.1001/jamanetworkopen.2022.0517)
Supplement: Supplement 1. — Trial Protocol [file jamanetwopen-e220517-s001.pdf]

Titel: Acupuncture vs. placebo acupuncture and vs. standard therapy for pain control after elective caesarean section - a pilot case series study and a randomized controlled trial

geplante Dauer 11/2014 – 12/2015

Leiter der Studie

PD Dr. T. Usichenko
Klinik für Anästhesiologie, Intensivmedizin, Notfallmedizin und Schmerzmedizin
Universitätsmedizin Greifswald
Sauerbruchstraße
D-17475 Greifswald
Telefon: 049-(03834) 865893
Telefax: 049-(03834) 865802
E-Mail: taras@uni-greifswald.de

Prüfärzte:

OA Dr. med. Th. Heße
Herr Bertold Henkel
Klinik für Anästhesiologie, Intensivmedizin, Notfallmedizin und Schmerzmedizin

Prof. Dr. A. Mustea
Klinik für Gynäkologie und Geburtshilfe
Universitätsmedizin Greifswald
Sauerbruchstraße
D-17475 Greifswald

Contents

| | | |
|---|---|---|
| | CONTENTS | 2 |
| 1. | GENERAL INFORMATION | 3 |
| 1.1 | Institution and investigators | 3 |
| 1.2 | Synopsis | 4 |
| 2. | SCIENTIFIC BACKGROUND AND AIMS OF THE STUDY | 5 |
| 2.1 | Background | 5 |
| 2.2 | Aims of the study | 6 |
| 3. | ETHICAL ASPECTS | 6 |
| 3.1 | Benefit-risk considerations | 7 |
| 3.2 | Protection of patients | 7 |
| 4. | STUDY DESIGN | 8 |
| 5. | PATIENTS' SELECTION | 8 |
| 5.1 | Inclusion criteria | 8 |
| 5.2 | Exclusion criteria | 8 |
| 5.3 | Discontinuation criteria | 9 |
| 6. | TREATMENT PLAN | 9 |
| 6.1 | Pilot study | 9 |
| 6.2 | Group allocation | 10 |
| 6.3 | Study procedure | 10 |
| 6.4 | Outcome measures | 11 |
| 7. | SAMPLE SIZE AND STATISTICS | 11 |
| 8. | REFERENCES | 12 |
| 9. | APPENDICES | |
| | Appendix A (Informed Consent Acupuncture) | 14 |
| | Appendix B (Informed Consent Quality Management System) | 15 |
| | Appendix C (Patient information) | 16 |
| | Appendix D (Standardized Spinal Anaesthesia | 21 |
| | Appendix E (Outcome Measures) | 22 |

# 1. GENERAL INFORMATION

## 1.1. Institutions and Investigators

Institutions:

Klinik für Anästhesiologie, Intensivmedizin, Notfallmedizin und Schmerztherapie
und
Klinik für Gynäkologie und Geburtshilfe
Universitätsmedizin Greifswald
Sauerbruchstraße
D-17475 Greifswald
Telefon: 049-(03834) 865893
Telefax: 049-(03834) 865802

Principal Investigator:

PD Dr. Taras Usichenko
Klinik für Anästhesiologie, Intensivmedizin, Notfallmedizin und Schmerzmedizin

Clinical Investigators:

Dr. Thomas Heße
Mr. Berthold Henkel
Klinik für Anästhesiologie, Intensivmedizin, Notfallmedizin und Schmerzmedizin
Prof. Dr. A. Mustea
Klinik für Gynäkologie und Geburtshilfe
Sauerbruchstr.
17475 Greifswald
E-Mail: mustea@uni-greifswald.de

## 1.2. Synopsis

| | |
|---|---|
| Title of the study: | Acupuncture vs. placebo acupuncture and vs. standard therapy for pain control after elective caesarian section - a pilot case series study and a randomized controlled trial |
| Study period: | 02/2015 – 05/2016 |
| Aim of the study: | To investigate whether acupuncture with indwelling fixed needles reduces pain and analgesic requirement as compared to placebo and standard therapy in patients after caesarian section (CS) |
| Design: | Pilot case series investigation with subsequent randomised controlled blinded clinical trial |
| Number of patients: | N = 180 |
| Inclusion criteria: | Adult patients scheduled to elective CS<br>Uncomplicated CS in spinal anaesthesia with <60 min. duration<br>Without previous opioid and psychotropic medication<br>Given informed consent |
| Outcome measures: | Postoperative analgesic requirement<br>Pain intensity<br>Incidence of side effects<br>Physiological parameters |

# 2. SCIENTIFIC BACKGROUND AND AIMS OF THE STUDY

## 2.1 Background

### 2.1.1 Postoperative pain relief

Despite advances in knowledge of pathophysiology, pharmacology of analgesics and the development of more effective techniques for perioperative analgesia, many patients continue to experience distressing pain after elective surgery (1,2). It is well known that inadequately controlled pain results in an increased postoperative morbidity, delayed recovery, a delayed return to normal daily living and reduced patient satisfaction (3). The standard method of pain control after major surgery – pharmacotherapy with opioid analgesics – is often accompanied with the side effects such as respiratory depression, decreased intestinal motility, nausea, vomiting and itching (4-6).

Postoperative pain (POP) therapy in patients after caesarian section (CS) often remains insufficient and can be improved (7). Recently, using the data from the annual audits within the quality management system (QMS) in POP treatment in our institution, we observed that the patients after CS reported more pain on movement than the patients after other surgical procedures: mean 6.1 vs. 4.2 (VRS-11; $p<0.0001$) (8). This can be explained by the fact that pharmacological treatment in the obstetrical wards worldwide (including our institution) is restricted due to peculiarities of this patients' category (7). POP treatment should not interfere with the mother's ability to care for her baby and should be free from adverse neonatal effects in breast-feeding women. Thus, only few analgesic drugs match these requirements and are recommended for postoperative analgesia after CS (7,9).

### 2.1.2 Acupuncture for postoperative pain control

Acupuncture is one of the non-pharmacological techniques, which can be used for complementary analgesia after surgery (10). During last decade AA became increasingly popular in perioperative medicine (11). In a recent systematic review Sun et al. found out that perioperative acupuncture allowed to reduce the consumption of postoperative opioid analgesics and reduced the intensity of postoperative pain. Also acupuncture was associated with a lower incidence of opioid-related side effects such as nausea, dizziness, sedation, pruritus and urinary retention (12). Using the acupuncture methods, described in the review and our own expertise in perioperative acupuncture (13,14), we applied body and ear acupuncture for treatment of POP in several patients scheduled for elective CS. The

promising results included the reduction of POP intensity and dose of analgesics as well as the increased satisfaction with POP treatment (15).

In the present project we are going to test the methodology of acupuncture analgesia after CS and generate the data to calculate the sample size for the subsequent randomised controlled trial (RCT). In RCT we aim to investigate the effectiveness of acupuncture added to standard therapy for pain control in patients after elective CS. We try to develop a protocol for a simple complementary method for acute pain relief, which can be easily applied in clinical routine by anaesthesiologists who have no prior knowledge on acupuncture.

## 2.2 Aims of the Study

### 2.2.1 Primary objective:

To investigate the effectiveness of acupuncture added to standard therapy for pain control in patients after elective CS.

### 2.2.2 Secondary objective:

To investigate whether acupuncture added to standard therapy for pain control also influences the side effects of standard postoperative analgesia in patients after elective CS.

# 3. ETHICAL ASPECTS

The present study protocol complies with the requirements of the following directives and guidelines:

- Declaration of Helsinki (48 General Assembly, Somerset West, South Africa, 1996)
- Good Clinical Practice in the Conduct of Clinical Trials on Medicinal Products for Human Use (Brussels, 20.06.96, E6GCPD12/01 May 96)
- Berufsordnung für die deutschen Ärzte (Dtsch Ärzteblatt 94, 2354 - 2363, 1997)
- Standards of the Department of Anaesthesiology, Intensive Care Medicine, Emergency Medicine and Pain Medicine, University Medicine of Greifswald

### 3.1 Benefit-risk consideration

The patients receiving acupuncture are expected to have benefit in reduction of pain intensity and analgesic requirement; thus a decrease in the rate of typical adverse effects of analgesics is hypothesised. The prognosis for the beneficial effect of acupuncture is based on the results of randomised controlled studies (10-15).

Since the body acupuncture will be performed using the indwelling permanent needles with 5 mm length, we did not expect the serious complications attributed to body acupuncture such as pneumo- and haematothorax and haemopericard (16). The patients will receive acupuncture in supine position, therefore the traumatic consequences of vasovagal reaction can be excluded (16). The most common side effects of auricular acupuncture, described in case reports, were endocarditis and perichondritis (16-18). Therefore, we shall exclude the patients at risk (with damaged or prosthetic cardiac valves) for endocarditis from our investigation. In cases of acupuncture-associated perichondritis the treatment including parenteral antibiotics and surgical intervention were described (18). We did not observe serious side effects of AA in more than 600 patients treated with indwelling fixed needles during the prospective clinical trials on analgesic effects of AA (19).

In this planned study the investigators will examine the indwelling fixed acupuncture needles on the day following surgery and the needles will be withdrawn on the 2nd postoperative day. The careful inspection along with the adequate disinfection of the skin before needle insertion enables the early detection and therapy of side effects and complications of acupuncture due to local inflammation and potential infection.

### 3.2 Protection of Patients

Ethics Committee

The protocol including the copies of the “Informed Consent Form” (**Appendix A & B**) and ”Special Information for Patients” (**Appendix C**) will be submitted to the Ethics Committee of the University of Greifswald. Written approval of the study protocol and the proposed “Informed Consent Form” (**Appendix A & B**) should be obtained before the start of the study.

Patient information

The patients will be informed both orally and in a written form about the study procedure, potential benefits and risks of the participation in the study, the right to withdraw from the study at any time, and to obtain written informed consent prior to any study procedures.

Insurance

The question of additional insurance, covering the costs of potential injuries due to various aspects of clinical trial, will be clarified with the Allianz Insurance Company.

## 4. STUDY DESIGN

This will be a prospective randomised, patient-blinded, placebo-controlled clinical study with non-randomised arm of standard therapy at one centre – the Department of Gynaecology and Obstetrics, University Medicine of Greifswald. The duration of the study will be 3 days for each patient. Local ethics committee should approve the design of the study. A total of one hundred and eighty patients scheduled for elective CS will be recruited according to patients' selection criteria.

## 5. PATIENTS' SELECTION

### 5.1 Inclusion Criteria

1. Patients with an American Society of Anaesthesiologists physical status of I to III scheduled for elective caesarean section in spinal anaesthesia
2. Surgery time does not exceed 60 minutes
3. Patients without previous opioid and psychotropic medication
4. Patients ranged 19-45 years old
5. Patients who have given written informed consent

### 5.2 Exclusion Criteria

1. Recidivist alcoholics
2. Local skin infection at the sites of acupuncture

3. Age < 19 and > 45 years
4. Switching to general anaesthesia in cases where spinal anaesthesia fails
5. Surgery time more than 60 minutes
6. Intraoperative complications (bleeding, required blood transfusion more than 6 units of packed cells, cardiovascular instability, required catecholamines except for short hypotension after induction of spinal anaesthesia )
7. Patients who consumed opioid medication at least 6 months before surgery
8. Patients who are unable to understand the consent form and fill in the study questionnaire
9. History of psychiatric disease

### 5.3 Discontinuation Criteria

1. In case of necessity to change the perioperative treatment scheme for particular patient
2. In case of local or systemic infection
3. In case of severe intercurrent disease
3. All patients are free to withdraw from participation in this study at any time, for any reason, specified or non-specified, and without penalty or loss of benefits to which the patient is otherwise entitled

## 6. TREATMENT PLAN

### 6.1 Pilot Study

A pilot case series investigation was performed to refine the methodology of acupuncture analgesia in patients after elective CS and to generate the data to calculate the sample size for the randomized controlled trial.

The retrospective data from the previous case series (15) were added to the data of 19 consecutive patients, who wished to receive acupuncture in addition to the pharmacological POP treatment according to internal standard of the University Medicine of Greifswald and were analysed. The results of the descriptive analysis (in particular pain intensity on movement as primary outcome measure) were used to calculate the sample size of the subsequent prospective RCT (s. Chapter 7). The patients signed the agreement about the scientific publication of their data from the pilot investigation.

## 6.2 Group Allocation

Following the signing of the informed consent for participation in this study (**Appendix A**) the study patients will be randomly allocated to receive acupuncture (60 patients) or placebo acupuncture (60 patients) in addition to standard therapy of POP. Randomisation will be performed using the method of sealed envelopes. Patients who will not wish to receive acupuncture will be asked to take part in a routine internal audit within the quality assurance activity on POP treatment (**Appendix B**) and thus will constitute the non-randomised arm of "standard therapy alone" in our investigation.

## 6.3 Study procedure

**Acupuncture and placebo intervention**

Four ear acupuncture points MA-SC, MA-TF1, MA-IC1 and MA-AT1 will be needled bilaterally in patients, assigned to acupuncture group. The choice of the ear acupuncture points is based on previously reported data about postoperative pain relief with AA after major surgery (19) and on the neurophysiological considerations (20). Disposable indwelling steel ear acupuncture New Pyonex needles, sized 0.2 x 1.5 mm and embedded in skin-coloured adhesive tape will be used for this procedure. For body acupuncture, LI4, ST36, SP6 and BL25-27 will be needled bilaterally using the indwelling intradermal 0.14 x 5 mm needles and will be attached with self-adhesive tape – New Pyonex placebo needles, originally designed for placebo control procedure.

The New Pyonex placebo needles will be used for placebo acupuncture intervention and will be applied to the same areas, as the real acupuncture needles, however without subsequent stimulation by the means of massage.

The acupuncture needles or placebo needles will be placed in the morning before surgery and retained *in situ* 3 days following the SC surgery. Before the needle insertion, the skin will be completely disinfected with alcohol swabs.

**Spinal anaesthesia and postoperative analgesia**

All study patients will receive a standardised spinal anaesthesia with 7.5 mg hyperbaric bupivacaine and 5 mcg sufentanil, which provides sufficient analgesia maximally 4 hours after the surgery. The treatment of POP after CS according to internal standard of University Medicine of Greifswald includes oral acetaminophen 4 x 1 g daily supplemented by

diclofenac 3 x 50 mg daily, if necessary. In case of insufficient analgesia, subcutaneous injections of 7.5 mg piritramide (opioid analgesic with 0.7 potency of morphine) are allowed up to 6 times daily **(Appendix D)**.

The patients will be instructed by the acupuncturist to stimulate acupuncture needles by the means of massage in case they feel the pain intensity higher than 4 measured using Verbal Rating Scale 11 (where 0=no pain; 10=maximal pain imaginable). The patients will be unaware of their allocation either to acupuncture or placebo acupuncture group. The success of patient's blinding should be tested at the end of the study.

### 6.4 Outcome Measures (Appendix E)

Primary outcome measure: pain intensity on movement taken on the 1$^{st}$ postoperative day

Secondary outcome measures:
-incidence of analgesia side effects
-the intensity of maximal and minimal pain after CS surgery
-the intensity of pain on movement on the 3$^{rd}$ postoperative day
-life quality items, disturbed by pain
-total amount of acetaminophen, diclofenac and piritramid
-time-points of mobilisation and withdrawal of Foley catheter
-patients' satisfaction with postoperative analgesia
-success of patients' blinding

## 7. SAMPLE SIZE AND STATISTICS

In order to calculate the appropriate sample size we set the level of significance to 0.05 and power at 90%. Based on the results of our pilot investigation and recently evaluated audit (8) and thus expecting to find a 30% difference in pain intensity on movement on the 1$^{st}$ day following surgery between acupuncture group and placebo group and 30% difference between placebo group and standard therapy group (with standard deviation 50% from mean value) the number of patients was calculated to be 55 per group (taking account the Bonferroni-adjustment for multiple comparisons among 3 study groups). Since the drop-out/withdrawal

rate may reach the 10-15% in clinical studies on postoperative pain relief with acupuncture (12,13), the sample size was set to a 60 patients per group and a total of 180 patients. Normally distributed continuous data will be compared using the Student's t-test and an analysis of variance, as appropriate. Skewed data will be compared using Kruskall-Wallis and Friedman test. Chi-square test will be used to analyse the success of patients' blinding and the incidence of analgesia-related side effects. SPSSStatistics Software for Mac (Version 22.0) will be used for data analysis.

## 8. REFERENCES

1. Apfelbaum JL, Chen C, Mehta SS, Gan TJ. Postoperative pain experience: results from a national survey suggest postoperative pain continues to be undermanaged. *Anesth Analg* 2003;97:534-40.
2. McGrath B, Elgendy H, Chung F et al. Thirty percent of patients have moderate to severe pain 24 hr after ambulatory surgery: a survey of 5,703 patients. *Can J Anesth* 2004;51:886-91.
3. Joshi GP, Ogunnaike BO. Consequences of inadequate postoperative pain relief and chronic persistent postoperative pain. *Anesthesiol Clin North America* 2005;23:21-36.
4. Schug SA, Zech D, Grond S. Adverse effects of systemic opioid analgesics. *Drug Saf* 1992;7:200-13.
5. Ballantyne JC, Carr DB, deFerranti S et al: The comparative effects of postoperative analgesic therapies on pulmonary outcome: cumulative meta-analyses of randomized, controlled trials. *Anesth Analg* 1998;86:598-612.
6. Jones EA, Bergasa NV. The pruritus of cholestasis and the opioid system. *JAMA* 1992;268:3359-62.
7. Lavoie A, Toledo P. Multimodal postcesarean delivery analgesia. *Clin Perinatol* 2013;**40**:443-55.
8. Hesse T, Julich A, Usichenko TI. Happy despite aching after caesarean section – an audit within the quality management system for postoperative pain. Under review in *International Journal of Obstetric Anaesthesia* 16 December 2014.
9. Siddik SM, Aouad MT, Jalbout MI, et al. Diclofenac and/or propacetamol for postoperative pain management after cesarean delivery in patients receiving patient controlled analgesia morphine. *Reg Anesth Pain Med* 2001;**26**: 310–5.

10. Wang SM, Kain ZN, White PF. Acupuncture analgesia: II. Clinical considerations. *Anesth Analg* 2008;106(2):611-21.
11. Chernyak GV, Sessler DI. Perioperative acupuncture and related techniques. *Anesthesiology* 2005;102:1031–1049.
12. Sun Y, Gan TJ, Dubose JW, Habib AS. Acupuncture and related techniques for postoperative pain: a systematic review of randomized controlled trials. *Br J Anaesth* 2008;101:151-60.
13. Usichenko TI, Dinse M, Hermsen M, Witstruck T, Pavlovic D, Lehmann Ch. Auricular acupuncture for pain relief after total hip arthroplasty – a randomized controlled study. *Pain* 2005;114:320-7.
14. Wetzel B, Pavlovic D, Kuse R, Gibb A, Merk H, Lehmann C, Wendt M, Usichenko TI. The effect of auricular acupuncture on fentanyl requirement during hip arthroplasty: a randomized controlled trial. *Clin J Pain* 2011;27:262-7.
15. Usichenko TI. Acupuncture as part of multimodal analgesia after caesarean section. *Acupunct Med* 2014;32(3):297-8.
16. Rampes H. Adverse reactions to acupuncture. In: Medical acupuncture-a western scientific approach. Ed: Filshie J, White A. Chirchill livingstone, 1998.
17. Lee and McIlwain Subacute bacterial endocarditis following ear acupuncture *Int J Cardiol* 1985;7:62-3.
18. Evans M. Litigation and ethical issues affecting acupuncture. *Acupunct Med* 1989;6:24-7.
19. Usichenko TI, Lehmann Ch, Ernst E. Auricular acupuncture for postoperative pain control: a systematic review of randomised clinical trials. *Anaesthesia* 2008;63:1343-8.
20. Usichenko TI, Mustea A, Pavlovic D. On ears and Head. *Acupunct Med* 2010;28:165-6.

# APPENDICES

## Appendix A

### Einverständniserklärung für die Anwendung der Akupunktur zur Schmerzlinderung nach einem Kaiserschnitt

Name, Vorname der Patientin:

Geburtsdatum:

Ich wurde vom unterzeichnenden Arzt über Art, Ablauf und Ziele der Akupunktur sowie die zu erwartenden Wirkungen, Nebenwirkungen und Risiken des Verfahrens aufgeklärt. Die spezielle Patienteninformation („Akupunktur zur Schmerzlinderung nach Kaiserschnitt“) habe ich gelesen und verstanden. Ich fühle mich umfangreich/ausreichend aufgeklärt. Es bestehen meinerseits keine weiteren Fragen bzw. Unklarheiten.

Meine Teilnahme an der Akupunktur-Studie ist freiwillig. Ich kann jederzeit, auch ohne Angabe von Gründen, die Mitwirkung/Mitarbeit beenden, ohne dass mir dadurch Nachteile entstehen.

Ich bin damit einverstanden, dass die im Rahmen der klinischen Beobachtung erhobenen Daten zur wissenschaftlichen Auswertung in pseudonymisierter Form verwendet werden.

Ich erkläre mich bereit, zu den obigen Bedingungen an dieser Behandlung teilzunehmen.

Greifswald, den Unterschrift der Patientin/des Patienten

Greifswald, den Unterschrift des aufklärenden Arztes:

**Appendix B**

## Einverständniserklärung für die Auswertung und Publikation der Ergebnisse des Qualitätsmanagements für die postoperative Schmerztherapie am Universitätsklinikum Greifswald

Name, Vorname der Patientin:

Geburtsdatum:

Ich wurde vom unterzeichnenden Arzt über Art, Ablauf und Ziel der Befragung aufgeklärt.

Ich bin damit einverstanden, dass die im Rahmen der klinischen Beobachtung erhobenen Daten zur wissenschaftlichen Auswertung in pseudonymisierter Form verwendet werden.

Greifswald, den Unterschrift der Patientin/des Patienten

Greifswald, den Unterschrift des aufklärenden Arztes:

**Appendix C**

**Patienteninformation**

**Studie über die Anwendung der Akupunktur für die Schmerzlinderung nach einem Kaiserschnitt**

Sehr geehrte Patientin,

bei Ihnen ist die Durchführung einer Kaiserschnitt-Operation zur Entbindung vorgesehen.

Wir möchten Sie einladen, an einer Studie zur **Akupunkturbehandlung** für die **Schmerzlinderung nach der Operation** teilzunehmen. Sie erhalten die Akupunktur **zusätzlich** zur üblichen Behandlung mit Schmerzmedikamenten.

Es ist bekannt, dass Akupunktur die Schmerzen nach Operationen lindert und damit den Einsatz von Schmerzmedikamenten verringert. Deshalb möchten wir diese Methode zur Linderung der Schmerzen bei Patientinnen nach dem Kaiserschnitt in unserer Klinik einführen. Dafür untersuchen wir in der vorliegenden Studie die genaue Wirksamkeit der Akupunktur.
Die Behandlung erfolgt **am Morgen vor der Operation** an bestimmten Punkten des Körpers und des Ohres (siehe Abbildung). Es werden dafür sehr dünne und kurze Akupunkturnadeln benutzt.

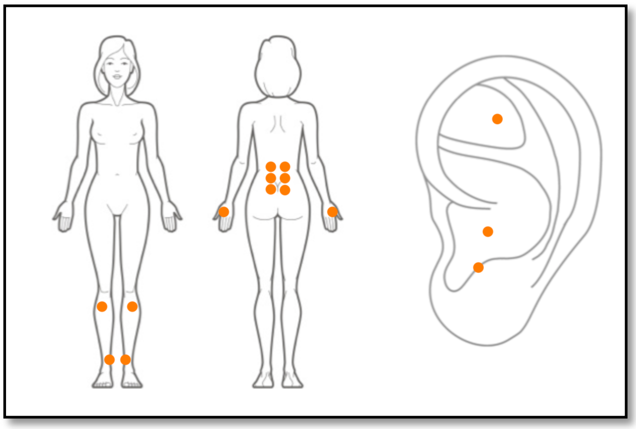

Da die Anwendung innerhalb einer Studie erfolgt, wird vor der Behandlung per Zufall entschieden, ob Sie der Akupunktur-Gruppe oder der Gruppe mit Placeboakupunktur zugeordnet werden. In jedem Fall erhalten Sie sowohl am ersten als auch am dritten Tag nach der Operation eine Visite durch einen Narkosearzt mit individueller Abstimmung Ihrer Schmerztherapie.

Wenn Sie sich für die Teilnahme an unserer Akupunktur-Studie entscheiden, werden wir Sie mündlich und schriftlich über alle weiteren Details informieren.
Wir bedanken uns bereits jetzt für Ihre Mitarbeit!

PD Dr. T. Usichenko
Dr. Thomas Heße
Berthold Henkel
Klinik für Anästhesiologie, Intensivmedizin, Notfallmedizin und Schmerzmedizin

**Appendix D**

## Spezielle Patienteninformation

Titel der Studie: **Akupunktur zur Schmerztherapie nach Kaiserschnitt**

**Leiter der Studie:** PD Dr. T.I. Usichenko
Universitätsmedizin Greifswald
Klinik für Anästhesiologie und Intensivmedizin
Ferdinand-Sauerbruch-Straße
17475 Greifswald

**Prüfärzte:** Dr. med. Thomas Heße
Herr Berthold Johannes Henkel

**Ort der klinischen Studie:** Klinik für Anästhesiologie und Intensivmedizin
Universitätsmedizin Greifswald
Ferdinand-Sauerbruch-Straße
17475 Greifswald
Telefon: 049-(03834) 865801
Telefax: 049-(03834) 865803
E-Mail: kai-sekretariat@uni-greifswald.de

### Einleitung

Sehr geehrte Patientin,

wir freuen uns darüber, dass Sie sich für die Anwendung der Akupunktur als zusätzliches Verfahren der Schmerzlinderung im Rahmen Ihres Kaiserschnittes entschieden haben.

Der Begriff „Akupunktur“ setzt sich aus zwei Worten zusammen: Acus = Nadel (lat.) und punger, punctum = stechen (lat.). Damit wird eine Heilmethode bezeichnet, bei der sehr feine, dünne Nadeln an bestimmten Punkten des Körpers oder des Ohres gesetzt werden. Die Auswahl dieser Punkte erfolgt durch den hierfür speziell ausgebildeten Therapeuten.

## Wie wirkt Akupunktur?

Durch verschiedene wissenschaftliche Studien konnte bereits bewiesen werden, dass die Akupunktur die Bildung und Freisetzung von schmerzlindernden und entzündungshemmenden Substanzen im Körper bewirkt. Besonders im Rahmen der Behandlung akuter und chronischer Schmerzen besitzt dieses Verfahren heutzutage einen viel versprechenden Stellenwert. Sowohl die Körper- als auch die Ohrakupunktur werden bei einer Vielzahl von Krankheitsbildern angewendet und auch bei größeren operativen Eingriffen steht die Methode aufgrund der o.g. Wirkungen im Mittelpunkt zahlreicher Untersuchungen.

## Vorgehensweise

In unserer Studie wollen wir wissen, ob die Anwendung der Akupunktur zusätzlich zur normalen Schmerzbehandlung zu einer weiteren Schmerzlinderung nach einem Kaiserschnitt führt.

Hierbei bilden wir zunächst 2 Patientengruppen. Bei den Patientinnen der ersten Gruppe führen wir am Morgen vor der Operation eine Akupunkturbehandlung an speziellen Akupunkturpunkten durch: 20 Akupunkturdauernadeln - je 1 an beiden Händen, je 2 an den Beinen, 6 am Rücken sowie je 4 an beiden Ohren. Patientinnen der zweiten Gruppe erhalten an den identischen 20 Punkten eine Placeboakupunktur.

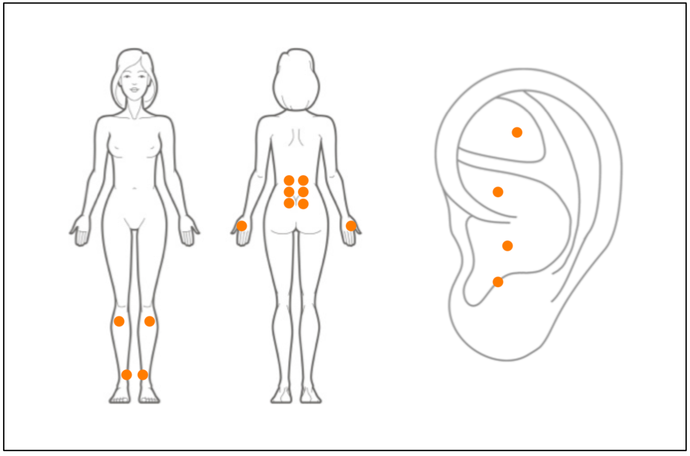

Die sehr dünnen und nur wenige mm langen Akupunkturnadeln sind durch Pflaster fixiert. Während und nach der Operation werden diese Akupunkturpunkte 3 mal täglich mittels manueller Massage stimuliert. Sie werden am Ende des 2. Tages nach der Operation entfernt.

Alle Patientinnen erhalten die übliche, in unserer Klinik bewährte medikamentöse Schmerzbehandlung nach einem Kaiserschnitt.

Wir wollen die Schmerzintensität und den Schmerzmittelverbrauch nach der Operation untersuchen. Sowohl am 1. als auch am 2. Tag nach dem Kaiserschnitt werden wir Sie deshalb in Ihrem Krankenzimmer besuchen, um Ihre Schmerzempfindungen und den Verbrauch an Schmerzmitteln zu erfragen.
Alle Patienten erhalten für den Kaiserschnitt eine Spinalanästhesie mit modernster Überwachung. Die Anästhesieführung erfolgt mit den aktuell zur Verfügung stehenden, üblichen Narkosemitteln, ohne dass für den Patienten zu irgendeinem Zeitpunkt ein zusätzliches, über das umfangreiche Aufklärungsausmaß hinausgehendes, Risiko besteht.

**Mögliche Nebenwirkungen der Akupunktur**

Die Akupunktur ist eine wirkungsvolle Therapie, die außerordentlich selten Nebenwirkungen hervorrufen kann. Der Einstich der Nadel bewirkt manchmal vorübergehend ein unangenehmes Gefühl. Weiterhin sind gelegentlich Kreislaufreaktionen wie Schwindel, Müdigkeit oder Kollapsneigung beobachtet worden.

Eine Infektion der Einstichstelle ist möglich, tritt jedoch ebenfalls sehr selten auf. Vorbeugend wirken hier die vorangehende Desinfektion der Haut und die Verwendung von sterilen Einmalnadeln. Geringfügige Blutungen nach dem Entfernen der Nadeln sind ebenfalls beschrieben. Allerdings wird dieser Blutung von vielen Akupunkteuren auch eine therapeutische Wirkung zugeschrieben. Es ist demnach fraglich, ob eine kleinere Blutung überhaupt zu den unerwünschten Wirkungen der Akupunktur zählt.

Zu jedem Zeitpunkt Ihres stationären Aufenthaltes können Sie, ohne Angabe von Gründen, Ihre Teilnahme an der Studie beenden. Es entstehen keinerlei Nachteile für Sie. Bei Rückfragen können Sie sich gern an uns wenden. Unter den folgenden Telefonnummern ist einer der Prüfärzte ständig in der Klinik zu erreichen:

**Tel. 03834/86-5893**

**Jede Information, die während des Verlaufs der Studie verfügbar wird und für Sie von Bedeutung sein kann, wird Ihnen durch das ärztliche Personal mitgeteilt.**

Wenn Sie die spezielle Patienteninformation gelesen und verstanden haben, wird Ihnen diese ausgehändigt. Zusätzlich werden Sie mündlich über alle Details der Studie informiert. Nach der Aufklärung unterschreiben Sie bitte die Einverständniserklärung, wenn Sie bereit sind, an der Studie teilzunehmen. Eine Kopie der unterschriebenen Erklärung wird Ihnen überlassen.

Vielen Dank für Ihre Mitarbeit!

PD Dr. Taras I. Usichenko
Dr. Thomas Heße
Berthold Johannes Henkel

**Appendix D**

**Spinalanästhesie zur Sectio caesarea – standardisiertes Vorgehen**

Prämedikation:

- keine
- bei Bedarf 150 mg Ranitidin am Vorabend sowie am Morgen der OP

intraoperatives Monitoring:

- 1-2 venöse Zugänge
- kontinuierliches Monitoring des Blutdrucks, der Herzfrequenz, der pulsoxymetrischen Sauerstoffsättigung und des Elektrokardiogramms sowie des Kardiotokogramms

Durchführung der Spinalanästhesie:

- Verwendung einer atraumatischen G25 Sprotte-Spinalnadel
- Punktion des Subarachnoidalraumes zwischen L3+L4 oder L2+L3 nach lokaler Infiltrationsanästhesie
- Injektion von 7,5mg hyperbarem 0,5%igem Bupivacain und 5µg Sufentanil in den Subarachnoidalraum
- Testung der Anästhesieausdehnung mit der Temperatur- und der pin-prick-Methode, Freigabe der Operation ab einem Anästhesielevel von mindestens T6

Therapie einer durch die Spinalanästhesiebedingten Hypotension

- Bei Eintreten einer Hypotension (RR systolisch < 100 mmHg oder RR-Abfall < 30% des Ausgangswertes) wird Akrinor® (Cafedrin/Theodrenalin) nach Wirkung intravenös bis zur Normotension titriert
- Eine Bradykardie (Herzfrequenz < 50/min) wird mit Atropin behandelt

Weitere Medikationen

- Jede Patientin erhält nach der Abnabelung des Kindes 3 IE Oxytocin sowie 3 g Ampicillin/Sulbactam intravenös

## Appendix E

Patienten-Nr.: ____________

# *Fragebogen zur postoperativen Schmerztherapie*

*(bitte diese Seite am 1. Tag nach dem Kaiserschnitt ausfüllen)*

Datum:

Geburtsdatum: |__|__|__|__|__|__|
Tag Monat Jahr

1. Wie stark war Ihr maximaler Schmerz seit der Operation bis jetzt?

| | | | | | | | | | | |
|---|---|---|---|---|---|---|---|---|---|---|
| 0 | 1 | 2 | 3 | 4 | 5 | 6 | 7 | 8 | 9 | 10 |

Kein Schmerz — Stärkster vorstellbarer Schmerz

2. Wie stark war Ihr minimaler Schmerz seit der Operation bis jetzt?

| | | | | | | | | | | |
|---|---|---|---|---|---|---|---|---|---|---|
| 0 | 1 | 2 | 3 | 4 | 5 | 6 | 7 | 8 | 9 | 10 |

Kein Schmerz — Stärkster vorstellbarer Schmerz

3. Wie stark sind Ihre Schmerzen bei Bewegung jetzt?

| | | | | | | | | | | |
|---|---|---|---|---|---|---|---|---|---|---|
| 0 | 1 | 2 | 3 | 4 | 5 | 6 | 7 | 8 | 9 | 10 |

Kein Schmerz — Stärkster vorstellbarer Schmerz

4. Hatten Sie nach der Operation folgende Symptome?

| | | |
|---|---|---|
| Übelkeit | Ja ☐ | Nein ☐ |
| Erbrechen | Ja ☐ | Nein ☐ |
| Müdigkeit | Ja ☐ | Nein ☐ |

# *Fragebogen zur postoperativen Schmerztherapie*

*(bitte diese Seite am Entlassungstag ausfüllen)*

1. Wenn Sie nach der Operation Schmerzen hatten, haben die Schmerzen folgende Bereiche beeinträchtigt?

| | | |
|---|---|---|
| Bewegung | Ja ☐ | Nein ☐ |
| Stimmung | Ja ☐ | Nein ☐ |
| Schlaf | Ja ☐ | Nein ☐ |
| Lebensqualität | Ja ☐ | Nein ☐ |

2. Wie stark sind Ihre Schmerzen bei Bewegung jetzt?

| 0 | 1 | 2 | 3 | 4 | 5 | 6 | 7 | 8 | 9 | 10 |
|---|---|---|---|---|---|---|---|---|---|---|

Kein Schmerz — Stärkster vorstellbarer Schmerz

3. Wie zufrieden sind Sie mit unserer Schmerztherapie?
(1 – sehr gut; 2 – gut; 3 – befriedigend; 4 – ausreichend; 5 – mangelhaft)

| 1 | 2 | 3 | 4 | 5 |
|---|---|---|---|---|

4. Ihrer Meinung nach haben Sie eine richtige oder eine Placebo-Akupunktur erhalten?

Ja ☐ Nein ☐ Weiß nicht ☐

5. Würden Sie sich bei einem erneuten Kaiserschnitt wieder für die Akupunktur entscheiden?

Ja ☐ Nein ☐

Herzlichen Dank für Ihre Unterstützung!
