# Supplementary material for: Effectiveness of Acupuncture for Pain Control After Cesarean Delivery: A Randomized Clinical Trial
Source: JAMA Netw Open. 2022 Feb 28;5(2):e220517. doi: 10.1001/jamanetworkopen.2022.0517 (PMC8886541; doi:10.1001/jamanetworkopen.2022.0517)
Supplement: Supplement 2. — eTable 1. Missing Data Across the Study Groups and End Points eTable 2. Mobilization and Foley Catheter Removal [file jamanetwopen-e220517-s002.pdf]

Supplementary Online Content

Usichenko TI, Henkel BJ, Klausenitz C, et al. Effectiveness of acupuncture for pain control after cesarean delivery: a randomized clinical trial. *JAMA Netw Open*. 2022;5(2):e220517. doi:10.1001/jamanetworkopen.2022.0517

eTable 1. Missing Data Across the Study Groups and End Points

eTable 2. Mobilization and Foley Catheter Removal

This supplementary material has been provided by the authors to give readers additional information about their work.

© 2022 Usichenko TI et al. *JAMA Network Open.*

eTable 1. Missing Data Across the Study Groups and Endpoints

**Missing data for following endpoints in each study group, No. (%)**	**Acupuncture n=60**	**Placebo n=60**	**Standard care n=60**
Maximal pain	1 (2)	4 (6)	0
Minimal pain	1 (2)	4 (6)	0
Pain on movement			
first postoperative day	1 (2)	4 (6)	0
day of discharge	2 (4)	5 (8)	2 (3)
Pain disturbed various items of life quality	2 (4)	5 (8)	4 (6)
Analgesics requirement	0	0	0
Analgesia-related side effects	1 (2)	4 (6)	0
Satisfaction with pain treatment [a]	2 (4)	5 (8)	2 (3)
Duration of hospital stay, days [a]	0	2 (4)	0
Mobilisation on both days	0	0	0
Foley catheter removed	0	1 (2)	0

© 2022 Usichenko TI et al. *JAMA Network Open.*

eTable 2. Mobilisation and Foley Catheter Removal

**Endpoints**	**Acupuncture Group (AG) n=60**	**Placebo Group (PG) n=60**	**Standard Care Group (SG) n=60**	**AG vs. PG**		**AG vs. SG**		**PG vs. SG**	
				**ES**	***P* Value**	**ES**	***P* Value**	**ES**	***P* Value**
Mobilisation, CS day, No. (%)									
none	19 (32)	41 (68)	48 (81)	2.13	.0003	3.58	< .001	1.68	> .99
sitting up	25 (43)	17 (29)	11 (17)	1.5	.024	2.5	.024	1.7	.6
standing up	13 (22)	0 (0)	1 (2)		< .001		.001		> .99
ambulation	3 (5)	2 (3)	0 (0)						
Mobilisation, 1. pO day, No. (%)									
none	0 (0)	2 (3)	0 (0)						
sitting up	0 (0)	0 (0)	10 (17)						
standing up	1 (2)	9 (15)	15 (25)						
ambulation	59 (98)	49 (83)	35 (58)	1.18	.012	1.69	< .001	1.43	0.03
Foley out, No. (%)									
CS day	2 (3)	0 (0)	0 (0)						
1. pO day	55 (93)	43 (72)	42 (70)	1.33	0.003	1.37	0.002	1.02	> .99
2. pO day	3 (5)	15 (25)	18 (30)						

Abbreviations: AG, acupuncture group; PG, placebo group; SG, standard care group; ES, effect size.
Data are absolute number of patients (%); *P*=statistical significance in Fischer's exact test after Bonferroni adjustment. Effect size calculated as relative risk.

© 2022 Usichenko TI et al. *JAMA Network Open.*
