# Supplementary material for: Effectiveness of Acupuncture for Pain Control After Cesarean Delivery: A Randomized Clinical Trial
Source: JAMA Netw Open. 2022 Feb 28;5(2):e220517. doi: 10.1001/jamanetworkopen.2022.0517 (PMC8886541; doi:10.1001/jamanetworkopen.2022.0517)
Supplement: Supplement 3. — Data Sharing Statement [file jamanetwopen-e220517-s003.pdf]

Data Sharing Statement

Usichenko. Effectiveness of Acupuncture for Pain Control After Cesarean Delivery. *JAMA Netw Open*. Published February 28, 2022. doi:10.1001/jamanetworkopen.2022.0517

Data
Data available: No

Additional Information
Explanation for why data not available: on request the anonymized data will be available via email of the corresponding author: usichent@mcmaster.ca
